# Evaluating the Impact of the National Health Service Digital Academy on Participants’ Perceptions of Their Identity as Leaders of Digital Health Change: Mixed Methods Study

**DOI:** 10.2196/46740

**Published:** 2024-02-21

**Authors:** Amish Acharya, Ruth Claire Black, Alisdair Smithies, Ara Darzi

**Affiliations:** 1 Institute of Global Health Innovation Imperial College London London United Kingdom

**Keywords:** digital leadership, professional identity, dissertation of practice

## Abstract

**Background:**

The key to the digital leveling-up strategy of the National Health Service is the development of a digitally proficient leadership. The National Health Service Digital Academy (NHSDA) Digital Health Leadership program was designed to support emerging digital leaders to acquire the necessary skills to facilitate transformation. This study examined the influence of the program on professional identity formation as a means of creating a more proficient digital health leadership.

**Objective:**

This study aims to examine the impact of the NHSDA program on participants’ perceptions of themselves as digital health leaders.

**Methods:**

We recruited 41 participants from 2 cohorts of the 2-year NHSDA program in this mixed methods study, all of whom had completed it >6 months before the study. The participants were initially invited to complete a web-based scoping questionnaire. This involved both quantitative and qualitative responses to prompts. Frequencies of responses were aggregated, while free-text comments from the questionnaire were analyzed inductively. The content of the 30 highest-scoring dissertations was also reviewed by 2 independent authors. A total of 14 semistructured interviews were then conducted with a subset of the cohort. These focused on individuals’ perceptions of digital leadership and the influence of the course on the attainment of skills. In total, 3 in-depth focus groups were then conducted with participants to examine shared perceptions of professional identity as digital health leaders. The transcripts from the interviews and focus groups were aligned with a previously published examination of leadership as a framework.

**Results:**

Of the 41 participants, 42% (17/41) were in clinical roles, 34% (14/41) were in program delivery or management roles, 20% (8/41) were in data science roles, and 5% (2/41) were in “other” roles. Interviews and focus groups highlighted that the course influenced 8 domains of professional identity: commitment to the profession, critical thinking, goal orientation, mentoring, perception of the profession, socialization, reflection, and self-efficacy. The dissertation of the practice model, in which candidates undertake digital projects within their organizations supported by faculty, largely impacted metacognitive skill acquisition and goal orientation. However, the program also affected participants’ values and direction within the wider digital health community. According to the questionnaire, after graduation, 59% (24/41) of the participants changed roles in search of more prominence within digital leadership, with 46% (11/24) reporting that the course was a strong determinant of this change.

**Conclusions:**

A digital leadership course aimed at providing attendees with the necessary attributes to guide transformation can have a significant impact on professional identity formation. This can create a sense of belonging to a wider health leadership structure and facilitate the attainment of organizational and national digital targets. This effect is diminished by a lack of locoregional support for professional development.

## Introduction

### Background

Delivering the digital transformation of the United Kingdom’s National Health Service (NHS) has been a long-standing aim. In the “What Good Looks Like” framework, by 2025, the NHS aims to have all integrated care systems and associated trusts reach core digital capability [1]. The key to this digital leveling-up strategy is the need to support professional development and training opportunities across integrated care systems [2]. To facilitate system-wide progress, there is a growing need for digitally proficient leadership teams; however, one of the main barriers identified by the NHS Transformation Directorate has been a lack of a “clear steer” for digital decisions [3].

Digital health resources and digital tools that were adopted through necessity during the COVID-19 pandemic have led to a paradigm shift in routine care. The scope of these resources has been significant across health care, including remote patient-clinician consultations and diagnostics [4-6]. However, as the health care service looks to enact facets of the *NHS Long Term Plan* and scale future sustainable digital change, possessing robust leadership to set this direction is key [7].

The NHS Digital Academy (NHSDA) designed its flagship course to deliver this support to emerging leaders. Each cohort of approximately 100 professionals is selected from applicants who are directly employed by the NHS or social care in England [8]. Digital health leadership is delivered in 2 accredited components. The first, resulting in a Postgraduate Diploma (PGDip) in Digital Health Leadership, uses a blended learning approach to provide a theoretical foundation for topics such as user-centered design [9,10]. This involves web-based teaching on 6 core modules structured around assessment deadlines including the essentials of health systems, implementing change, health information systems, user-centered design, actionable data analytics, and leadership change. Subsequently, students can undertake a 1-year Master of Science (MSc) degree. The MSc degree uses a dissertation of the practice model, where students focus on practicable applications of theory within defined digital transformation projects. This self-directed period of study involves candidates’ leading projects within their own host organizations with periodic deadlines to guide progress and continued access to the support of the teaching faculty. In this manner, the course facilitates a workplace-based learning model geared toward supporting students to use research to solve a real-world problem in their organization. Although the course has been shown to effectively impact the attainment of national digital priorities [11], little is known about the effect on participants’ perceptions of themselves as digital leaders or their professional identity.

Defining professional identity is difficult owing to a lack of standardization of the term. It has been associated with knowledge acquisition, performance of typical tasks, displays of expected behaviors, or shared ethos and value systems [12]. In other contexts, professional identity involves the integration of the personal and professional selves [13]. A scoping review by Cornett et al [14] identified 5 constructs associated with health professional identity, including lived experience (eg, practicing), the world around me (eg, the workplace), belonging (eg, collective identity), me (eg, self in relation to the profession), and learning (eg, acquiring skills). However, the review examined health professional practice and did not specifically examine digital leaders [14]. Understanding what constitutes the identity of a digital health leader is potentially more problematic, given that the field is relatively nascent. Consequently, understanding what knowledge is needed, the tasks or behaviors that are expected of leaders, and what constitutes core values is likely to remain ill-defined until the digital health landscape has evolved. Furthermore, an individual’s perception of their professional role is a dynamic process and can be augmented by one’s context [15]. Determinations regarding the extent to which one feels like a professional can therefore be difficult to ascertain.

Despite these challenges, professional identity formation has been shown to be an increasingly important aspect of learning development. Within clinical settings, professional identity contributes to the delineation of practice boundaries as well as avoiding confusion regarding individuals’ roles within wider teams [16]. With a growing body of clinicians involved in digital leadership, this is particularly important, as studies have demonstrated that doctors can often encounter difficulties when reconciling managerial and clinical responsibilities. Moreover, aspects of professional identity, such as “belongingness,” have been associated with greater workforce retention [17]. Therefore, there is a growing drive to evaluate how courses and educational curricula impact an individual’s identity.

### Aim

This mixed methods study aimed to understand the influence of the NHSDA Digital Health Leadership program on participants’ perceptions of themselves as digital health leaders. This will facilitate a greater understanding of the core values associated with digital leadership and provide insights to improve courses globally.

## Methods

This study was conducted as a mixed methods study involving a web-based questionnaire, interviews, and focus groups.

### Recruitment

Participants in the first 2 cohorts of the NHSDA’s flagship Digital Health Leadership program were recruited for the study. All participants had completed both years of the program and were >6 months from completion to avoid recency bias. This could involve overemphasizing the impact of later teaching in course compared to that which occurred earlier. Studies suggest that a later evaluation can provide a more holistic evaluation [18]. It also provided time for candidates to reflect on future career opportunities. No other exclusion criteria were placed upon participants; therefore, a nonprobabilistic sampling method to reach the necessary sample size was used. Eligible participants were contacted through email by a member of the research team (AA) with no direct link to the NHSDA. Both cohorts were impacted by the COVID-19 pandemic, particularly with respect to their dissertation projects that were undertaken during the pandemic.

### Scoping Questionnaire

A previously validated web-based scoping questionnaire was used to provide insights and feedback on the course [11]. This questionnaire explored the impact of the course on the development of facets such as “social intelligence,” “interpersonal skills,” and “courage.” It also examined the effect of the course on future goals, asking “Would you consider any of the following additional training options in Digital Health Leadership or a related field within the next 2 years?” This questionnaire was developed to map specifically onto the NHSDA program objectives and encompassed questions including individuals’ perspectives on development and digital leadership. It also sought to ascertain feedback on the aspects of the course that were most influential on participants. A total of 2 authors (RCB and AA) developed the survey questions, whereas a third (AS) independent author was involved to discuss disagreements. The participants were recruited via an email containing an anonymous link. The links were delivered to all eligible individuals separately from the program to avoid selection and response biases based on prior performance in the course.

### Semistructured Interviews

Following the survey, anonymous responses were quantitatively (multiple-choice questions) and qualitatively analyzed (free-text sections). Themes derived from the analysis were elicited by 2 authors (RCB and AS). The results from the survey were then used to develop the question guides for interviews and focus groups (Multimedia Appendix 1). A third author (AS) was involved to help resolve disagreements. Interviews were designed to gain a more in-depth understanding of individuals’ perceptions of the values and skills associated with digital health leadership and how the course has influenced these areas. Enrollment to an interview was not dependent on completion of the survey or prior performance. All interviews were conducted web-based via Microsoft Teams (Microsoft Corporation). AA conducted all the semistructured interviews, and AA had no formal role within the NHSDA and no prior interaction with any participants to avoid response biases.

### Focus Groups

To ascertain the shared experience of participants and paralleling the collaborative learning approach used by the program, web-based focus groups were also undertaken using the Microsoft Teams platform. In addition, the focus groups examined the participants’ contrasting experiences of the course. A total of 3 focus groups were conducted, with the facilitator (AA) not being affiliated with the NHSDA. Each focus group involved 4 to 5 participants. The invitation to participate in the focus groups was not contingent on the completion of any previous phase of the study. As with the interviews, the focus groups used open-ended prompts to foster responses. In addition, the facilitator encouraged open discussion between participants. Identity involves the development of attributes congruent with the profession, that is, a common set of values about what it means to be a digital health leader [19]. By facilitating focus group discussions regarding how digital leadership is perceived, its underpinning principles, and how one can develop the necessary skills to become a more effective leader, a greater understanding of these shared values was attained.

### Analysis

Survey responses were collated through the web-based tool Qualtrics (Qualtrics International Inc). Qualtrics automatically aggregates replies and provides frequencies from the respondents by choice. Given the small number of responses and because the initial survey was used to inform further study phases, no statistical analysis was undertaken. Free-text options were inductively thematically analyzed until data saturation was achieved by an author (AA) and validated by another (RCB). Both qualitative and quantitative responses were used to inform the development of the topic guides following discussion between the authors. Specifically, the authors focused on areas of disagreement or if a particular topic recurred across the responses of different participants.

Audio recordings, obtained with the consent of participants for both interviews and focus groups, were transcribed using the web application Descript (Descript, Inc). The accuracy of the outputs was confirmed by one author (AA), who was present in the interviews and focus groups. Anonymized transcripts were then uploaded to the analysis tool MAXQDA (VERBI GmbH). A deductive thematic analysis was conducted using a technique previously used in similar studies [20]. This involved familiarization with the transcripts by 2 authors (RCB and AA). The transcripts were then coded with the data explored to examine the frequency and relationship of the codes. Similar codes were combined into themes and subthemes, which were aligned with the components of professional identity elicited by Chin et al [19]. This review was selected as a framework on which to base the thematic analysis for 3 reasons: first, because of its comprehensive evaluation of identity with 10 evidence-based facets described; second, the examination of internship or workplace-based learning parallels the educational model of the NHSDA’s second year; and finally, the authors’ examination of how these components map to other contexts can facilitate cross-discipline comparisons was helpful in understanding the participant’s identity across wider teams [21]. Although Chin et al [19] found that only a subset of these components was applicable to higher education internships, this study examined the relevance of all components, as some were more significant in postgraduate studies.

As a means of validation, the anonymized transcripts were reviewed again, and themes were amended until a consensus was attained. All discrepancies within the coding exercise or allocations of themes were discussed until resolution. Themes that were consistently mentioned by different participants, those that aligned with findings from the questionnaire or focus group, and those that were regarded as stronger determinants were considered more impactful influences. A constructivist approach was used as the basis of this study, which paralleled the active learning undertaken throughout the program. The paradigm focuses on the importance of active learning and its transformation through experience [22,23]. It involves the engagement and reflection of the learner, which can be impacted by context, knowledge, motivation, values, or organizational setting [24]. This is particularly pertinent to identity development, which can be influenced by such intrinsic and extrinsic factors.

High-scoring dissertations across the 2 included cohorts were also evaluated independently by 2 authors (RCB and AA). The authors then mapped the skills exhibited in these manuscripts to the components of professional identity. Students were required to make explicit reference to a particular component for it to be mapped. The authors discussed any disagreements until a consensus was reached.

### Ethical Considerations

Approval to conduct this study was provided by the Institutional Review Board at Imperial College London (reference EERP2021-026a). All participants provided explicit written consent to participate in the study and were free to withdraw at any time. No participant received financial remuneration for being involved in the study. All data including transcripts and survey data were kept anonymous, in keeping with the secure data storage policies of Imperial College London.

## Results

### Overview

A total of 41 eligible participants completed the web-based survey, of which 42% (17/41) were female and 59% (24/41) were male. Most participants were in clinical health care roles (17/41, 42%), whereas 34% (14/41) were in program delivery or management roles; 20% (8/41) were in informatics or data science roles, and 5% (2/41) were in “other” roles. Of those surveyed, 59% (24/41) reported that the NHS Digital Academy course had a strong and direct impact on their working practice, 27% (11/41) reported some impact, and only 2% (1/41) reported no effect. In total, 4 key themes were elicited from the inductive analysis of the free-text sections: transformative impact, valuing collaboration, goal setting, and improving positive perceptions. The selected results are presented in Multimedia Appendix 1.

**Figure 1:**
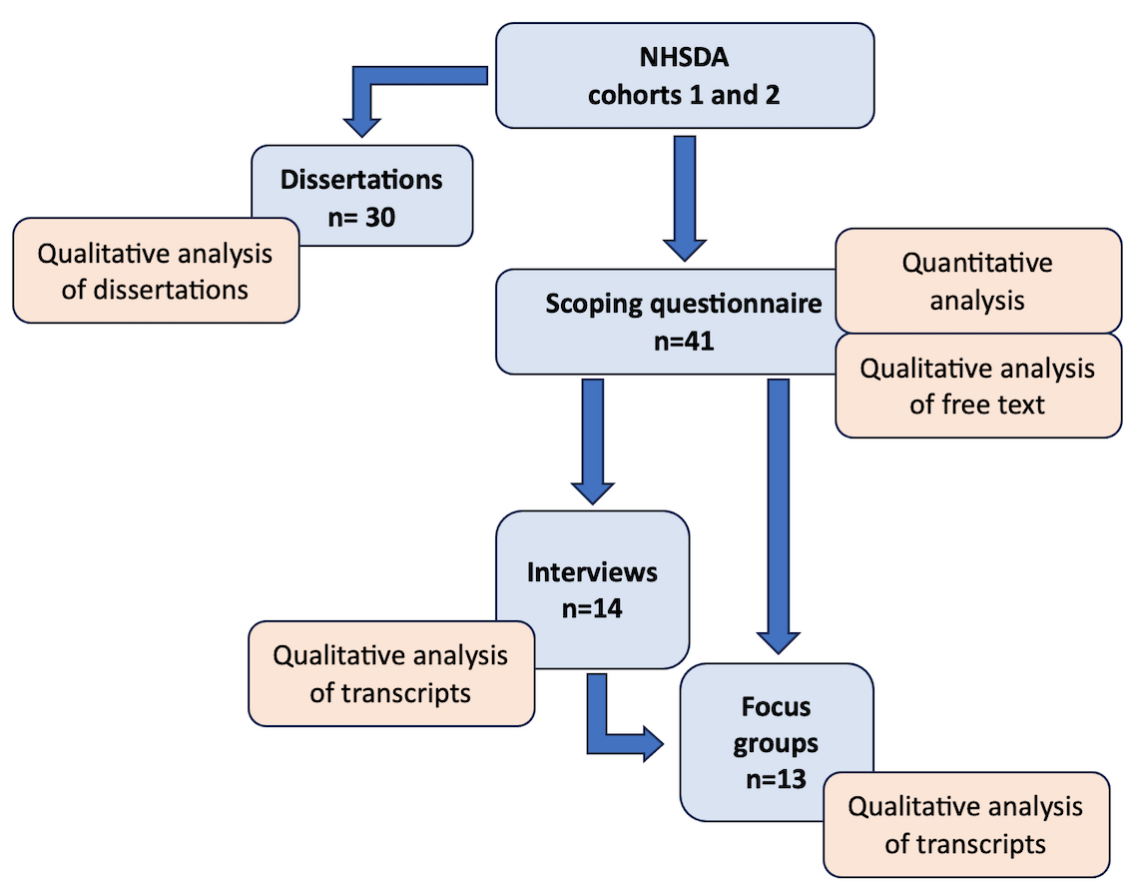
The flow of participants in the study.

Semistructured interviews were conducted with 34% (14/41) of participants. The demographics of which paralleled those from the wider cohort, with 43% (3/7) of participants identifying as female and 50% (7/14) working in clinical roles. In total, 3 focus groups were held with more than half of the attendees (7/13, 54%) not involved in the preceding interviews. The data sources including the number of participants used in the study is presented in Figure 1.

Thematic analysis mapped findings from interviews and focus groups to 8 of the 10 components of professional identity highlighted by Chin et al [19]. Internship experience was not measured, as most of the cohort had been in their roles before the NHSDA and could not be considered entering an internship. However, aspects encompassing skill acquisition during dissertations were covered in other domains. The work environment was also not included, as the participants came from disparate fields, precluding comparisons. However, the findings were mapped to the following domains: commitment to the profession, critical thinking, goal orientation, mentoring, perception of the profession, socialization, reflection, and self-efficacy. When undertaking the thematic analysis and mapping of the highest-scoring dissertation to the components from the framework by Chin et al [19], only 4 were found to be applicable. These included critical thinking, goal orientation, mentoring, and reflection. This is likely because the dissertation was more descriptive of a specific transformation project rather than reflective of the attitudes of participants toward digital health leadership as a whole.

Table 1 demonstrates how the course impacted these areas through quotations from the respondents.

**Table 1:** Key domains^a,b^ of professional identity with quotes from participants on the impact of the course.

Domain	Definition	Quote
Commitment to profession	The physical, mental, and emotional commitment to being a digital leader. Understanding the aims of leadership and demonstrating a willingness to achieve them.	“People saw what I was learning from the course and my enthusiasm for my career...I was invigorated and it encouraged me to do the best in my career path.” [Interviewee 13] “Having gone through the course, I want to pursue careers in digital in some fashion...it’s shifted the course of my career, I’m aspiring to national level roles.” [Interviewee 7]
Critical thinking	A metacognitive skill to critically evaluate the current standard and elicit new solutions. Involves understanding one’s own role as a digital leader and how one would want it to be.	“There is a critical language element to it [digital leadership]...you have more critical analysis aspect to your work...it [the National Health Service Digital Academy] has changed the way I approach and think about problems.” [Interviewee 6] “I became more strategic in my approach... it’s broader...it’s about thinking right...we need it think from top to bottom.” [Interviewee 5]
Goal orientation	How one achieves and defines the specific outcomes associated with being a digital leader. Involves having a conducive environment for task mastery and development as a digital leader.	“My perception has shifted...I see myself as a facilitator of digital transformation...I aim to maintain virtual delivery and my organization is helping me meet that need.” [Interviewee 11] “One of the most valuable things from the digital Academy...it made me understand where to make change, improve processes, how to measure that change and feeding it back...it is the core of our aims.” [Interviewee 1]
Mentoring	Acting as a mentor and having mentorship. Involves role modeling, feedback strategies, and encouraging self-reflection as a digital leadership, as well as a conducive work environment for mentor.	“I believe in paying it forward, I’ve brought back what I’ve learnt to building my informatics team.” [Interviewee 7] “I became very invigorated by the community...I spent an afternoon with a module lead in user design... then the head of user design centre in the NHS offered me an opportunity to shadow them.” [Interviewee 13]
Perception of the profession	Ideas about what it is to be a digital leader, the skills required, and its place in wider health care infrastructure.	“The NHSDA meant I didn’t hold those people on a pedestal...it [digital leadership] is not about having all the technical knowledge, it’s being able to pull together everyone toward the solution.” [Interviewee 11] “It has been transformational...just in the knowledge it has given me...on understanding the role...where it fits into organizational strategy...the scope.” [Interviewee 4]
Professional socialization	A sense of belonging to the wider community and being accepted as part of a group. Includes credentialing and peer networks.	“It has a level of kudos...there is good recognition that it, the academy skilled them up.” [Interviewee 12] “Now I’ve got a network of probably 100 or more contacts nationally...I would go and talk to them and say you must know someone locally who does this, any chance you could put me in touch?” [Interviewee 8]
Reflection	Reflecting on knowledge, cognition, professional identity, maturity, and the sense of professionalism within digital leadership. Involves ideas regarding professional development.	“It’s highlighted the positives and the negatives of my leadership style, my digital knowledge and also where I fit within an organization and nationally. So it’s given me that sort of self-awareness.” [Interviewee 10] “It helped me become better leader. It helped me understand how would I help people in the organization transform and be more innovative.” [Interviewee 2]
Self-efficacy	Self-belief or belief in one’s own capabilities to perform as a digital leader. Includes imposter syndrome and the impact of external opinions upon one’s own beliefs.	“When I first went on the digital academy...it felt like we were interlopers...throughout it continued to build my confidence levels and where I fit as a digital leader locally.” [Interviewee 14] “It made me think that I am a leader...I would never have applied for that Royal College job without it.” [Interviewee 3]

^a^Work environment not included as relevant components covered in “mentoring,” and participants came from disparate environments.

^b^Internship experience was not included. The participants represent the existing digital health leadership whose roles would not include internship. Areas of skill-building are covered within other domains.

### Commitment to the Profession

The program appeared to influence individuals’ commitment to digital health leadership. In total, 59% (24/41) of the cohort reported changing their roles following the course. Among the participants who changed their roles following the course, 46% (11/24) reported that the program had a strong impact on this decision. Inductive thematic analysis of the survey comment elicited this transformative impact of the course upon candidates’ careers, with several describing “life-changing” or “career-changing” effects. In one focus group, one informatician mentioned that “I do now feel like a leader, and I wasn’t going to stay in that organisation.” This new commitment led them to “find somewhere else that I [they] could be a digital leader.” Others reported that they “were looking at influencing policy, in a way I [they] hadn’t before...because of the course.” This commitment to digital health leadership has led them to apply for chief clinical informatics officer (CCIO) roles. The course also appeared to reaffirm participants’ motivation for undertaking digital health leadership roles. One CCIO stated:

[The course] hasn’t necessarily given me all the technical skills...but it’s greater than that. It’s given the background of how we’ve got to where we are now and inspired me to change things going forward.

### Critical Thinking

Critical thinking, which involves understanding a context and deriving new solutions, was found to be fostered predominantly through the MSc dissertation. As presented in Table 2, all but 3 of the 30 highest-ranking dissertation topics across the 2 cohorts involved critical analysis.

Providing a supportive environment for change enabled candidates to put theoretical learning into practice. An interviewee said “I [they] approach things differently, I’m [they are] more strategic, more constructed after the project.” These cognitive skills have continued postgraduation with individuals feeling they have “different tools that were picked up during the academy, which I [they] use day-to-day.”

**Table 2:** Topics of the highest-scoring dissertations of the 2 cohorts and components of identity that were incorporated.

Dissertation topic	Critical thinking	Goal orientation	Mentoring	Reflection
BYOD^a^ policy design and development for NHS^b^ Trusts		✓		✓
Board level digital readiness	✓	✓	✓	
Blueprint for digital excellence in the development of a new hospital	✓	✓	✓	✓
Implementing recommendations of Topol review		✓	✓	
Impact of digital working on patient care	✓	✓		✓
Improving performance of a cardiorespiratory outpatient department	✓	✓		✓
App to reduce suicide and self-harming and improve safety and clinical outcomes in mental health	✓	✓		✓
Standards and processes for sharing data across platforms and organizations	✓	✓		
Blueprint for digital first GP^c^	✓	✓	✓	✓
Participant preferences for contact and clinical research study enrollment	✓	✓	✓	✓
Evaluating impact of digital maturity on effectiveness and efficiency of care in adolescent inpatient mental health units	✓	✓		✓
Digital transformation of epilepsy care and monitoring	✓	✓		✓
Implementing SNOMED-CT^d^ coding into an EHR^e^ for clinical decision support, data sharing and medical pathway transformation	✓	✓	✓	✓
Direct web-based advice from consultant psychiatrists to GPs	✓	✓	✓	✓
Returning health professionals living with cancer to work via a digital resource	✓	✓	✓	✓
Enabling effective and appropriate use of virtual consultations with adolescents in psychiatry specialty settings	✓	✓		✓
An impact analysis of Morse system implementation and mobile device use by health visitors in rural Scotland	✓	✓	✓	
Optimizing remote access to primary care during COVID-19: a focus on patients with moderate to severe mental health needs	✓	✓		✓
Making quite voices louder: addressing health inequalities for people with moderate to severe mental health illness	✓	✓		✓
Digitally enabling primary care beyond the COVID-19 pandemic		✓	✓	
The impact of digital tools and ways of working on staff burnout and enjoyment of work in psychiatry	✓	✓	✓	✓
Optimizing culture of collaboration and learning to tackle health inequalities: a study of digital health Canada	✓	✓	✓	✓
Digital delivery: the future of UK diabetes education	✓	✓		✓
Impact of the implementation of a critical care information system on patient-facing clinical staff in an intensive care unit during the COVID-19 pandemic	✓	✓		✓
The key components of organizational culture for a digital first strategy	✓	✓	✓	✓
The relationship between funding and the digital maturity of NHS provider organizations	✓	✓		✓
Partnership between health care provider organizations and industry in adopting AI^f^ into health care practice	✓	✓		
A framework for effective prioritization of digital transformation projects in recently merged secondary care organizations	✓	✓		✓
Best practices for digital inclusion in at risk pediatric populations	✓	✓	✓	✓
Transformation at pace and scale by EPR^g^ sharing among high and low digitally mature hospital systems	✓	✓	✓	✓

^a^BYOD: bring your own device.

^b^NHS: National Health Service.

^c^GP: general practitioner.

^d^SNOMED-CT: Systematized Nomenclature of Medicine Clinical Terms.

^e^EHR: electronic health record.

^f^AI: artificial intelligence.

^g^EPR: electronic patient record.

### Goal Orientation

Goal orientation encompasses defining and accomplishing the specific outcomes of digital leadership within the NHS. This may involve achieving national priorities outlined in health policies, such as the *NHS Long Term Plan* or locoregional transformation targets. As the MSc model was designed to provide a supported environment to undertake these projects, it was unsurprising that all the dissertations evaluated involved an element of goal orientation. These projects varied from digitizing diabetes education platforms and booking processes to projects focusing on the implementation of the recommendations of the Topol review [25]. An interviewee said, “The reason I [they] chose this MSc project was because...it was my day job.” This pragmatic approach helped align the goals of the course with those of digital leaders. Survey comment analysis also elicited “goal setting” as a key theme. Several candidates identified future opportunities for further professional development across a broad range of areas, including policy development, finance, teaching, and strategy.

### Mentoring

The influence of the NHSDA on the provision and reception of mentorship was variable. Only 27% (11/41) of the survey respondents felt they acquired mentoring skills; however, 59% (24/41) reported that they were more able to develop capabilities within their teams. Moreover, 50% (15/30) of dissertations reflected the provision of mentoring within participants’ local organizations. Following attendance at the NHSDA, some candidates were encouraged to “develop the professional training development with my [their] own teams,” with a common theme being “paying forward” the knowledge they had acquired. Furthermore, the program provided opportunities for candidates to receive mentorship or “shadowing opportunities.” One candidate who developed an interest in user-centered design “spent a really impactful afternoon with a module lead” and, subsequently, connected with designers from NHS England. This culminated in a career change to a health care–based user experience department. These experiences were significantly influenced by candidates’ work environments, with others noting they were “still alone in the organization...with little guidance from management.”

### Perception of the Profession

An informatician reported, “since the course...I see myself [themselves] as a facilitator of digital transformation,” as opposed to their previous notions regarding a more technical role. This was echoed by others who perceived digital leaders as change agents: “not just somebody with the skills...but the ability to make connections to bring about transformation.” For more junior candidates, the course also helped level the hierarchy within the digital ecosystem. Having previously put “digital leaders on a pedestal” and believing that becoming one “was an unachievable target,” following the course, they believed that “it [being a digital leader] is not about having all the technical knowledge but being able to pull everyone together toward a solution.” Conversely, more established digital leaders had constructed their perceptions of digital leadership before the NHSDA, with 1 CCIO explaining, “it [the course] hasn’t changed the way I perceive what I do, it has made me more effective.”

### Professional Socialization

Socialization was a key untaught component of the course. In the questionnaire, 46% (19/41) reported that the MSc program influenced their feelings of socialization within digital leadership. Inductive thematic analysis of the survey elicited “valuing collaboration” as a common theme among respondents. Many were reporting that they now found value in a “network of like-minded professionals” and wanted to “understand [their] colleagues better.” Moreover, most respondents highlighted that the program taught them how to maintain effective relationships (29/41, 71%) and inspired a shared purpose among colleagues (30/41, 73%), both facilitating a common sense of belonging. A participant suggests the “main impact of the MSc was this community of leaders who understand transformation...and share knowledge with each other.” This “collaboration is helping me [them] realise they were no different.” This network facilitated wider professional socialization by providing participants with “incredible peer support” as well as “recognition within the wider community” of the NHSDA.

### Reflection

Reflection upon practice was a core facet of the dissertation, with candidates actively encouraged to examine their own practice and how it correlates with their perceptions of digital health leadership. Therefore, 80% (24/30) of the dissertations demonstrated evidence of reflective practice. This encouragement to reflect upon practice has led to several candidates reporting the academy “highlighted the positives and the negatives of my [their] leadership style,” fostering a “sort of self-awareness.” Reflection was also associated with candidates refining their perceptions of the nature of digital transformation. One CCIO from cohort 1 notes the NHSDA “makes you reflect on how we embark on this challenge of having to scale digital at pace in the context of the pandemic.”

### Self-Efficacy

The development of self-efficacy was found to be a key tenet of the program, with 61% (25/41) of the respondents reporting that the course had positively increased their confidence in their role. One candidate noted that “When I [they] first went on the digital academy there was an element of imposter syndrome,” being told, “not to think about imposters, you need to think as pioneers.” Others had reflected that the digital academy had given them “the confidence to lead in digital” and “empowerment...to recognize that I [they] have the ability to do anything I [they] put my [their] mind to.” This has led to several candidates being recognized as leaders within the wider digital health ecosystem, but not necessarily in their own organizations. One clinician noted that they “had taken up a few national unpaid roles”; however, another noted that “they [the director] was not interested...did not recognize the training we had.”

## Discussion

### Principal Findings

This study is one of the first to demonstrate the impact of a focused program on digital health leadership on attendees’ professional identity. The findings demonstrate that the course has a diverse range of impacts including commitment to the profession, critical thinking, goal orientation, mentoring, perception of the profession, socialization, reflection, and self-efficacy. By using a dissertation of the practice model, in which students undertake a supported digital transformation project, participants are provided with an opportunity to develop metacognitive and reflective skills. The effect of this skill development lasts beyond the course, with several participants altering their leadership style and developing more agile and collaborative approaches. Furthermore, the projects enable participants to define and attain digital goals, which may have been more difficult to define, benchmark, and achieve previously. The program reaffirmed attendees’ commitment to being or becoming a digital health leader, leading to more than half of the participants changing their roles after graduation. Among the group of individuals that changed roles, almost half noted that their experience within the NHSDA had a significant impact on this decision. In addition, the program dispelled the imposter syndrome felt by emerging leaders by increasing their confidence and a sense of professional belonging. This was facilitated by the network of alumni, which may help mitigate the organizational isolation felt by some participants.

Professional identity formation has become the focus of a diverse range of fields, including medical education [26]. Among health care professionals, studies have shown that the development of a shared core value set can have substantial benefits, including improving the well-being and resilience of physicians [27]. Professional identity’s influence in other areas is less well documented, but some benefits may be appreciable across a range of disciplines. In a study by Meadows and de Braine [28], industry leaders displayed stronger leadership identities during the COVID-19 pandemic to help overcome challenges such as the implementation of new technologies. Digital health leadership teams who were faced with comparable issues are likely to have also relied on stronger leadership identities during this time. Consequently, there is increasing interest in understanding how educational programs can foster professional identity in their cohorts. Some have suggested that to develop identity requires departing from traditional pedagogy and using greater participatory or sociocultural learning opportunities [29]. The NHSDA program uses a mixed approach, blending didactic learning with collaborative work in the first year and a dissertation of the practice model in the second year. Therefore, a breadth of impact, both intended and unintended, upon the identity of participants as digital health leaders were noted.

A principle focus of the course and the dissertation project is on reflective practice. Therefore, it was not surprising that 80% (24/30) of the projects incorporated these skills. Reflective exercises are an important component of professional identity and can help leaders hone their metacognitive and inductive reasoning skills. Studies have shown that through these processes, learners can also identify their own cognitive biases and avoid errors [30]. Moreover, the dissertation was also noted to foster critical thinking, with several participants reporting that they had become more “strategic.” Critical thinking is a higher-level cognitive skill in which individuals understand phenomena through their interpretation and inference of contributory factors and variables. Critical thinking enables learners to become more agile [31]. Given that the digital health landscape is continuing to evolve in the United Kingdom following the pandemic and the continued challenge of resource allocation, an ability to acclimatize to these newer contexts would appear integral. In fact, when asked directly “What is a digital health leader?” several respondents referred to this adaptability, noting the need for “fearlessness, curiosity, and being comfortable going into unknown territories.”

One of the key unintended consequences of this course has been its impact on professional socialization. Socialization is crucial for emerging learners to learn the values and beliefs necessary to succeed within their roles as well as to form a robust idea of what constitutes a digital health leader. The peer support, or “sphere of networking,” that has developed among participants has facilitated not only knowledge sharing but also a sense of a community of digital health leaders. Several participants refer to a sense of confidence and validation of their identity as they were able to collaborate with recognized digital leaders. This socialization is seen in other areas of health professional development and provides a sense of “belonging,” as well as facilitating transition across clinical roles (eg, clinician to leader) [14]. This may mitigate the varying support that participants receive within their organizations.

On the other hand, few participants reported being mentored, and many participants felt unrecognized within their local institutions. Mentorship is a crucial facet of identity, as it enables the observation, modeling, and imitation of leadership behaviors, as described by the social learning theory [32,33]. Consistent with previous studies, time pressures and competing demands are often barriers to mentoring in health care environments. Moreover, several participants reported a lack of recognition by their local management teams following the course. This lack of external validation as an emerging leader in digital health may have thus contributed to this shortage of mentorship opportunities. However, having engaged in the collaborative environment of the NHSDA, participants were more open to facilitating the future training of more junior members of their own teams. In addition, these local barriers may underpin the drive to find different opportunities and explain the high rates of role switching after graduation. Future work should look to examine these findings as well as how accreditation from courses such as the NHSDA can impact organizational buy-in.

### Limitations

However, these findings must be considered within the limitations of the study. Despite using a robust approach, the respondents represent a subsection of the eligible cohorts involved. Moreover, only high-scoring dissertations were evaluated, which may have skewed our findings. However, this decision was made because scores were given based on the comprehensiveness of the write-up not the quality or results of the project. Therefore, they provided a more detailed impression of the elements of professional identity included. These selection effects were mitigated by delivering the questionnaire widely, and not all perspectives could be explored. This may affect the generalizability of the results, but it does provide a strong indication of the breadth of influences of the course. Furthermore, as previously mentioned, there is no set definition of what it is to be a digital health leader. As such, components from other contexts have been used to frame this study, which may mean that certain nuances have been omitted. Although the use of a previous extensive systematic review reduced the likelihood of this, it cannot be considered comprehensive. Furthermore, both cohorts enrolled undertook at least part of their study during the COVID-19 pandemic, in which there was a significant change in the delivery of health care and the need for digital solutions [34]. The influence of these changes on participants’ experience of the course or its impact on their professional identity cannot be ascertained. Future work should examine what constitutes a digital health leader and how this differs from health leadership more generally. This could potentially result in defining a core value set to facilitate the evaluation of digital and clinical leadership courses. This examination would need to consider the technical and nontechnical aspects of digital health leadership, as understanding both facets is essential as digital transformation continues to accelerate.

### Conclusions

The increasing demand for clinical management to guide the next stages of transformation efforts requires a digitally adept corps of health leadership professionals. These digital leadership proficiencies must not only encompass technical skillsets but also include the values, judgments, and cultural beliefs about what it is to be a digital leader. The NHSDA and similar courses are likely to impact this identity formation through a broad range of effects, including socialization and professional commitment. However, further work is needed to understand what attributes are needed by a digital health leader so that training courses can be iterated and adapted. Moreover, this categorization will support the recognition of potential digital leaders who can be mentored within their local organizations, and key barriers to this progression can be overcome.

## Appendix

Multimedia Appendix 1 Survey results and interview topic guide.

## Abbreviations

CCIO: chief clinical informatics officer
PGDip: Postgraduate Diploma
MSc: Master of Science
NHS: National Health Service
NHSDA: National Health Service Digital Academy

## Data Availability

The data that support the findings of this study are available from the authors, but restrictions apply to the availability of these data, which were used under license for this study and thus are not publicly available.
