# Supplementary material for: Evaluating the Impact of the National Health Service Digital Academy on Participants’ Perceptions of Their Identity as Leaders of Digital Health Change: Mixed Methods Study
Source: JMIR Med Educ. 2024 Feb 21;10:e46740. doi: 10.2196/46740 (PMC10918534; doi:10.2196/46740)
Supplement: Multimedia Appendix 1 [file mededu_v10i1e46740_app1.docx]

*Survey Results*

Which of this best describes your primary job role? (Please specify if other)

Which, if any, of these leadership elements did the NHSDA course impact?

Which, if any, of the following change/innovation components did the course impact?

As a result of the course have you changed jobs, or is a change of role anticipated within the next 6 months?

If you have changed jobs, to what extent did the course impact upon your job change?

*Exploring key themes in the NHS Digital Academy PG Diploma and MSc*

Interview Protocol

The study will include individual interviews lasting approximately 45 minutes each. Individual interviews will be conducted remotely using Teams and will consist of one participant. Interviews will take place after the survey is closed. It is expected that most participants will have completed the survey but this is not a requirement. To limit researcher impact and bias, an individual not affiliated with the NHSDA will facilitate the groups as he does not have existing relationships with the study participants.

1.  Welcome

Welcome and thank you for volunteering for this study. I recognise that you are a busy professional and your participation is greatly appreciated.

Your participation in this individual interview will contribute to our evaluation study, exploring themes and quality factors in the PG Diploma in Digital Health Leadership, and its follow-up MSc dissertation module.

You have been asked to participate because I am interested in your experiences of the NHS Digital Academy.  Your participation in this study has the potential to inform the curriculum of the programme, which will benefit the experiences of future participants.

2.Introductions

I’ll start it off… my name is [*I will introduce myself and explain my role at IGHI and my role in the study*]. An assistant facilitator may also be present, who will introduce themselves and explain their role.

3.  Anonymity and right to withdraw

- The discussion will be audio and video-recorded, via Microsoft Teams but I’d like to assure you that the discussion will be anonymised.

- After the discussion, the recordings will be transcribed and analysed. Unanonymised recordings are transcribed/anonymised as soon as possible and then deleted with the anonymised transcripts stored securely according to Imperial College protocols (for 10 years after last use or publication).

- The recording files will be kept safely on a secure server and will be kept in accordance with research governance policies.

- During this study, you will be assigned a participant number so will therefore remain anonymous.

- I would also like to remind you that you have the right to withdraw from the study at any time with no penalty to yourselves. A withdrawal letter has been supplied, which can be completed and returned should you choose to.

- If you do not wish to answer a question, you do not have to.

4.  Ground rules

- The events we are focusing on here are limited to your experiences studying on the Postgraduate Diploma in Digital Health Leadership. I only want you to describe *how it was for you* at the time, what you did, what you learnt from your experience on the Digital Academy programme.  Remember, I am only interested in your personal experiences and reflections.

- You are requested to respect the confidentiality of others present in the group.

- Everything that you say in this room will be anonymised. While we will be reporting the findings in general, your personal anonymity will be maintained.

- There are no right or wrong answers, everyone’s experiences and perspectives are valid.

Finally, do you have any questions you would like to ask me about this study before we begin?

5. Your NHS Digital Academy experiences

The interview is designed to further explore three key themes and areas of the study

1. What is a digital health leader?
2. What are the most impactful aspects of the MSc and how have they influenced you as a digital leader? [priority question]

Prompts for follow up:

- 1. Do you work/approach things differently?
  2. Prompt: Has your perception of your role changed?
  3. Prompt: Have you changed roles?
  4. Prompt: Have responsibilities changed?
  5. Prompt: If yes, did the MSc influence the change?
  6. Prompt: How has the MSc influenced your personal future goals?

1. How has your experience in the MSc impacted your identity as a digital health leader in your organisation? [priority question]
   1. Prompt: Do you see national impact as a result of your project?
2. What critical skills, competencies, attitudes and values did the MSc help you develop to fulfil your role as a leader, and more generally?
   1. Prompt: Career, identity development, life goals, leadership development, confidence, what are you going to do next ….
3. How has the project impacted your perception of digital health leader?
   1. Do you have any future career directions you are planning how has the NHSDA impacted these
   2. What leadership skills did it help you develop
   3. What role did the project have in your development as a digital health leader

6. Next steps

Thank you for participating. I think this has been a very successful discussion.

Your descriptions of experiences and suggested educational recommendations have made a valuable contribution to the study.

7.Conclusion

I would like to remind you that any comments featured in this report will be anonymous. Thank you for your participation in this study.
